# Is 6 Weeks Postpartum Too Late for Contraceptive Counseling in the U.S.? A Look into the Timing of Postpartum Intercourse

**DOI:** 10.1080/00224499.2025.2585378

**Published:** 2025-11-17

**Authors:** Evangeline Warren, Fernanda L. Schumacher, Lisa Keder, Megan Lawley, Peggy Goedken, Amy C. Dupper, Maria F. Gallo

**Affiliations:** a The Ohio State University College of Medicine; b Division of Biostatistics, The Ohio State University College of Public Health; c Department of Obstetrics & Gynecology, The Ohio State University College of Medicine; d Department of Obstetrics & Gynecology, Emory University School of Medicine; e Division of Epidemiology, The Ohio State University College of Public Health; f Department of Epidemiology, University of North Carolina School of Global Public Health

## Abstract

Healthcare providers in the United States typically recommend that postpartum people wait 6 weeks before resuming vaginal sex to ensure time to recover from childbirth. Guidelines recommend that postpartum patients attend a 6-week postpartum visit, and this visit typically includes contraception provision and counseling. We analyzed data from a clinical trial of immediate postpartum use of injectable contraception on lactation, which followed patients from delivery through the first postpartum year. We calculated the prevalence of early resumption of vaginal sex, defined as having vaginal sex before 6 weeks postpartum. We also used bivariable and multivariable models to identify correlates of early resumption. Among the sample of 137 adult postpartum patients, 18.4% reported early resumption of vaginal sex; among this subgroup, the mean time to resumption was 3.9 weeks (standard deviation, 0.9 weeks). The sole factor evaluated that appeared to be correlated with early resumption of vaginal sex was education; college graduates had 70% lower adjusted odds of reporting early vaginal intercourse compared with those who had less education (*p* = .04). Our study findings suggest that resuming sex before 6 weeks postpartum is not rare and could place people at risk of a rapid repeat pregnancy.

## Introduction

Although the American College of Obstetricians and Gynecologists recommends continuing postpartum care across the “fourth trimester,” 6 weeks marks the traditional timing of the postpartum checkup in the United States (American College of Obstetricians and Gynecologists [ACOG], 2018). The 6-week visit often includes contraceptive counseling and method provision, if not already initiated independently by patients or in consultation with providers immediately postpartum, to mitigate risks of short interpregnancy intervals. As many as half of all women who commence intercourse before the 6-week benchmark do so without the use of contraception (Sok et al., 2016). Given that short interpregnancy intervals are associated with multiple adverse pregnancy and childbirth outcomes (Conde Agudelo et al., 2006; Wendt et al., 2012), a better understanding of the factors associated with the resumption of vaginal intercourse before the 6-week appointment is warranted.

Limited recent research has addressed early resumption of intercourse in the United States. In addition to Sok et al. (2016), who reported 43% of their participants had resumed vaginal intercourse by 6 weeks postpartum, Knutson et al. (2022) reported 9.9% of their participants had resumed intercourse within one month postpartum after first birth. A recent systematic review and meta-analysis specifically drawing on articles examining postpartum return to intercourse identified only two studies based in the United States (Abebe Gelaw et al., 2024), one of which surveyed women 6 months postpartum and did not report more granular timing of the resumption of vaginal sex (Brubaker et al., 2008) and the other of which focused on sexual activity 8–10 weeks postpartum (Yee et al., 2013). A separate systematic review examining sexual behavior during pregnancy and the postpartum period identified seven U.S.-based studies that included data on postpartum sexual behavior (Jawed-Wessel & Sevick, 2017). The studies included in the review were retrospective in nature, without specific questions in the immediate postpartum period contemporaneously documenting the return to intercourse. In the present study, we examined potential demographic and reproductive health correlates of resumption of vaginal sex before 6 weeks postpartum. The study sample was limited to postpartum adults who had delivered a singleton, term infant of normal weight without substantial health issues, thereby eliminating the potential confounding effect of many pregnancy and childbirth complications on the return to intercourse (Jawed-Wessel & Sevick, 2017).

## Method

We analyzed data from a parent study (*N* = 167) that enrolled labor and delivery patients at one of two study sites immediately following childbirth and followed them for one year. The parent study evaluated the effect of injectable contraception on lactogenesis and has been reported elsewhere (Gallo et al., 2025). Participants in the parent study either received an injectable contraceptive prior to hospital discharge, a placebo injection prior to discharge, or no injection prior to discharge. All participants received condom counseling and all participants affirmed both that they did not desire pregnancy in the 12 months following delivery and that they did not plan to use alternative hormonal contraceptives following delivery (Gallo et al., 2025).

We analyzed potential correlates measured at enrollment, consisting of standard sociodemographic factors: age (linear); race (collapsed to white vs. person of color due to small cell sizes); and college degree attainment. We also evaluated factors that, based on existing research (Abebe Gelaw et al., 2024; Hicks et al., 2004; Jawed-Wessel & Sevick, 2017; Lewis et al., 2010), we hypothesized could affect timing of resumption of sex: mode of delivery (vaginal or cesarean); live birth before the index birth (yes vs. no); and cohabitation (yes vs. no).

Our outcome of interest consisted of early resumption of vaginal sex, which we defined as occurring before 6 weeks postpartum and which we derived from two follow-up survey questions that asked (1) whether the respondent had resumed vaginal sex since delivery and, if so, (2) the timing of the resumption in weeks since delivery. We relied on a series of follow up surveys to construct this variable. We excluded participants with missing data for resumption of sex (*n* = 24) or for the date of resumption (*n* = 2), participants whose only follow-up response was less than 6 weeks postpartum and did not indicate they had resumed sex (*n* = 3), and participants whose reported timing was internally inconsistent (*n* = 1). This resulted in a final analytic sample of 137 respondents. The first follow-up survey was fielded one month (4 weeks) postpartum and 14 respondents indicated they had resumed vaginal sex at that time. Subsequent follow-up surveys occurred at 2-, 4-, 6-, 9-, and 12-months postpartum. For participants who did *not* indicate an early resumption of vaginal sex at one-month postpartum, we queried their next nonmissing survey response to determine their return to intercourse (most often the 2-month postpartum survey). An additional 11 respondents reported they had resumed intercourse less than 6 weeks postpartum in their later follow-up surveys, 51 respondents reported they resumed vaginal sex at or after 6 weeks, and 61 respondents reported they had not resumed vaginal sex after 6 weeks postpartum. The Ohio State University Institutional Review Board approved the study with a reliance agreement in effect for the secondary site, thereby ceding review and oversight to the Institutional Review Board at Ohio State.

We first analyzed the independence of the outcome of interest (early resumption of vaginal sex) and individual categorical correlates of interest using marginal logistic regression models. We then used multivariable logistic regression to analyze the relationship between the demographic factors and early resumption of vaginal sex separately from the reproductive factors to identify potential moderating variables (demographic model). Finally, we modeled the relationship of the demographic factors and reproductive health factors to our outcome of interest in a combined multivariable logistic regression model (combined model).

## Results

The sample was racially diverse, with over half of our participants identifying as a person of color (Table 1). Overall, 18.4% of participants reported early resumption of vaginal sex, and among this subgroup, the mean time to resumption of vaginal sex was 3.9 weeks postpartum (range, 2.0–5.5, standard deviation [SD], 0.9 weeks).

In the marginal analyses, none of the potential correlates were statistically significantly related to early resumption of vaginal sex (Table 2). In the demographic model, we found that, when adjusting for age, race, and cohabitation, participants who held a college degree had 73% lower odds of resuming vaginal intercourse earlier than 6 weeks compared to those with less education (*p* = .03). In the combined model, adjusting for demographic factors and factors related to pregnancy and delivery (mode of delivery and previous live birth), lack of a college degree remained a consistent correlate of early resumption of vaginal sex, with college graduates having 70% lower odds of reporting early vaginal intercourse (*p* = .04).

## Discussion

A substantial fraction of people (18.4%) in our trial reported resuming sex before 6 weeks postpartum. This finding is consistent with earlier evidence indicating that, despite healthcare providers commonly recommending a 6-week delay in the return to sex following delivery, early resumption is common (Abebe Gelaw et al., 2024). The robust association between the lack of a college degree and an early return to vaginal sex present in our data suggests that a college degree may be a proxy for health knowledge (E. M. Lawrence, 2017), socioeconomic status (Braveman et al., 2005), or reproductive autonomy (Grace & Anderson, 2018; Tarzia & Hegarty, 2021). This early return to vaginal sex, correlated with the absence of contraceptive use, may result in additional, unplanned pregnancies that are further correlated with economic pressures on the family unit, exacerbating inequalities between college-educated and non college-educated parents.

An individual might choose to delay a return to vaginal intercourse for several reasons. While there are not universal recommendations for the timing of resumption of sex, providers might recommend that the birthing person wait until bleeding stops, the cervix closes, and any tears have time to heal to reduce the risk of infection before engaging in any form of penetrative sex. In addition, sexual difficulties are common in the postpartum period (Cattani et al., 2022) and those experiencing sexual problems might often delay resumption of sex (Abebe Gelaw et al., 2024; Jeong et al., 2021; Serati et al., 2010). Similarly, the immediate postpartum period is often a time of decreased libido with birthing people needing time to physically recover from childbirth (Abebe Gelaw et al., 2024; Jeong et al., 2021; Serati et al., 2010), adjusting to the demands and stress of parenting an infant (Lawrence et al., 2007), navigating postpartum body image issues (Serati et al., 2010), reacting to changes in the relationship with a partner following childbirth (Lawrence et al., 2007; Schlagintweit et al., 2016), and experiencing postpartum depression (Jeong et al., 2021; Van Der Woude et al., 2015). Separately from clinical recommendations and practice, the 6-week benchmark has strong cultural support in the United States (Harrison et al., 2022; Serati et al., 2010).

Conversely, individuals may seek to resume vaginal intercourse in the immediate postpartum period in keeping with their high pre-delivery sexual desire and low sexual distress levels (Tavares et al., 2023), because their sexual desire had returned to predelivery levels (Ibrahim et al., 2025), or because their partner’s sexual desire was largely unaffected by the sociobehavioral experiences of the postpartum period (Hipp et al., 2012; van Anders et al., 2013). In addition, some patient-facing clinical websites now state that patients may resume intercourse as soon as they feel comfortable, stating only that patients should ensure any vaginal tears have healed (Mayo Clinic Staff, 2024).

The primary limitation of this study was our small sample size. Our null findings regarding pregnancy sequence and mode of delivery are consistent with null findings in other research on postpartum sexual difficulties (Cattani et al., 2022). Our null findings regarding race, however, are anomalous and may reflect the constraints of our small sample size and subsequent collapsing of nonwhite racial identities. Given the parent study’s focus on injectable contraceptives, some participants in this study commenced contraception prior to leaving the hospital after delivery. Because of the parent study’s design, only 28% of participants knew they had received an injectable contraceptive prior to discharge. In sensitivity analyses, the inclusion of this variable (whether participants knew they had received an injectable contraceptive) in our models had no effect on the significance, magnitude, or direction of our explanatory variables of interest. In addition, we tested for independence and found there was not a significant relationship between early resumption of penetrative sex and contraceptive status naivete, *X^2^*(1, *N* = 137) = .277, *p* = .599. In other words, participants with knowledge of contraceptive protection through injectable contraceptives were no more (or less) likely to return to penetrative sex before 6 weeks.

## Conclusions

We build on a nascent line of research examining patterns in postpartum intercourse amongst pregnant people in the United States. Our findings have significant implications for the importance of predelivery contraceptive counseling and the timing of contraceptive initiation postpartum, especially for pregnant people without a college degree. Among participants who returned to vaginal sex less than 6 weeks postpartum (18.4%), the mean timing to resumption was within 1 month of delivery, well before the traditional postpartum checkup. Clinicians should be mindful that a significant portion of their patients will have resumed vaginal sex before their first postpartum visit after hospital discharge and, as a result, should ensure that patients have access to contraceptive methods well in advance of this time. Separately, much of the previous literature identified a return to vaginal intercourse less than 6 weeks postpartum incidentally and retrospectively. Going forward, researchers should be mindful that surveys or interviews fielded after the 6-week mark are likely to miss contemporaneous reporting of return to intercourse and, therefore, might risk memory bias or desirability bias in the data.

## Figures and Tables

**Table 1.** Participant characteristics (*N* = 137), by intercourse resumption date.

	At least 6 weeks (*N* = 112)	Less than 6 weeks (*N* = 25)

Age (years)	29.0 (5.3)	27.6 (4.8)
	No. (%)	
*Race*		
White	50 (44.6%)	14 (56.0%)
Person of Color	62 (55.4%)	11 (44.0%)
*Live births*		
Index birth only	48 (42.9%)	7 (28.0%)
Previous live birth	64 (57.1%)	18 (72.0%)
*Mode of delivery*		
Vaginal	77 (68.8%)	19 (76.0%)
Caesarean section	35 (31.3%)	6 (24.0%)
*Educational attainment*		
Less than college degree	58 (51.8%)	18 (72.0%)
College degree or higher	54 (48.2%)	7 (28.0%)
*Cohabiting with father of index infant*		
Yes	88 (78.6%)	22 (88.0%)
No	24 (21.4%)	3 (12.0%)

**Table 2.** Associations between demographic and pregnancy factors and early resumption of intercourse following delivery (*N* = 137).

	Marginal models	Demographic model	Combined model

	OR (95% CI)	aOR (95% CI)	aOR (95% CI)
*Race*			
White	*Ref.*	*Ref.*	*Ref.*
Person of Color	0.63 (0.26, 1.52)	0.41 (0.15, 1.16)	0.36 (0.12, 1.10)
Age	0.95 (0.87, 1.03)	0.97 (0.88, 1.07)	0.95 (0.86, 1.06)
*Educational Attainment*			
Less than College Degree	*Ref.*	*Ref.*	*Ref.*
College Degree or Higher	0.42 (0.16, 1.08)	0.27* (0.08, 0.86)	0.30* (0.10, 0.96)
*Cohabitation w/Father of Index Infant*			
Not Cohabiting	*Ref.*	*Ref.*	*Ref.*
Cohabiting	2.00 (0.55, 7.29)	2.35 (0.61, 9.06)	2.25 (0.59, 8.54)
*Pregnancy Sequence*			
First Live Birth	*Ref.*		*Ref.*
Previous Live Birth	1.93 (0.74, 5.00)		2.23 (0.81, 6.12)
*Mode of Delivery*			
Vaginal	*Ref.*		*Ref.*
Cesarean Section	0.70 (0.25, 1.90)		0.88 (0.29, 2.71)

Exponentiated coefficients; 95% confidence intervals in brackets.

* *p* < .05.
